# A Report of Two Uncommon Cases of *Mycobacterium chelonae* with Localized and Disseminated Skin and Soft Tissue Infection

**DOI:** 10.3390/idr17010013

**Published:** 2025-02-08

**Authors:** Libardo Rueda Prada, Marko Gorasevic, Tatjana Gavrancic, Aayushi J. Rajani, Jason C. Sluzevich, Sangeeta Nair-Collins, Ravi V. Durvasula

**Affiliations:** 1 Division of Hospital Internal Medicine, Mayo Clinic, Jacksonville, FL 32224, USA; 2 Research Trainee, Mayo Clinic, Jacksonville, FL 32224, USA; 3 Medical College Baroda, Vadodara 390001, Gujarat, India; 4 Division of Dermatopathology, Mayo Clinic, Jacksonville, FL 32224, USA; 5 Division of Infectious Diseases, Mayo Clinic, Jacksonville, FL 32224, USA

**Keywords:** *Mycobacterium* infections, Nontuberculous, *Mycobacterium chelonae*, skin diseases, infectious

## Abstract

**Background**: *Mycobacterium chelonae* is a ubiquitous, rapidly growing, nontuberculous mycobacteria that primarily affects immunocompromised patients. The most common presentation is an atypical, chronic skin and soft tissue infection. Due to its high resistance rate, early diagnosis based on clinical suspicion, risk factor assessment, and exposure history is crucial for initiating appropriate multi-drug treatment. **Methods**: We report two cases of *M. chelonae* skin and soft tissue infections, one presenting with localized disease and the other with disseminated involvement. One case had a specific exposure to fish-related activities, a risk factor more commonly associated with *Mycobacterium marinum* infections rather than *M. chelonae*. **Results**: One of the cases involved osteomyelitis and tenosynovitis which are rare presentations of *M. chelonae* infection. While the limbs are the most commonly affected sites in disseminated *M. chelonae* infections, involvement of the lower extremities, as seen in one of our cases, is rarely reported. Treatment posed challenges due to antibiotic resistance and patient tolerance. However, in both cases where follow up was possible, prolonged multi-drug therapy led to complete resolution of the lesions. **Conclusions**: These cases highlight the importance of considering *M. chelonae* in chronic skin and soft tissue infections, especially in patient with relevant exposures or immunosupression. Uncommon presentations require a high index of suspicion. Given the challenges of treatment resistance and patient tolerance, prolonged multi-drug therapy remains essential for successful outcomes.

## 1. Introduction

*Mycobacterium chelonae*, *Mycobacterium abscessus*, and *Mycobacterium fortuitum* represent 80% of the 70 recognized rapidly growing mycobacteria (species that grow in laboratory media within 7 days) that affect humans [1,2]. *M. chelonae* is considered a ubiquitous nontuberculous mycobacteria (NTM) that is highly drug-resistant, which makes it a challenge to treat. It is broadly found in the environment (soil, dust, natural or treated water), plants, animal reservoirs (reptiles, fish, birds), and within free-living amoebae [2]. NTM form biofilms, making it difficult to eradicate even with standard disinfectants. Up to 83% of the city water in hemodialysis centers in the US contains NTM [3].

While *M. chelonae* infections are more commonly seen in immunocompromised patients, osteomyelitis, tenosynovitis, and disseminated diseases, including diseases affecting the lower extremities, have been rarely reported.

## 2. Case Presentation

### 2.1. Case 1: M. chelonae Tenosynovitis and Osteomyelitis

A 36-year-old female with a past medical history of type 1 diabetes mellitus, simultaneous kidney and pancreas transplant in 2017 complicated by the failure of both grafts (on maintenance prednisone 10 mg daily), now on hemodialysis presented to the emergency room (ER) for evaluation of recalcitrant right-hand infection of 2 months duration without improvement to oral antibiotics. She did not report any preceding trauma. She initially developed a red and painful papule on the distal interphalangeal joint of her right index finger, which did not respond to amoxicillin clavulanate, doxycycline, and topical mupirocin. She reported being an avid freshwater aquarium keeper and cleaning a fish tank without using protective gloves.

**Figure 1:**
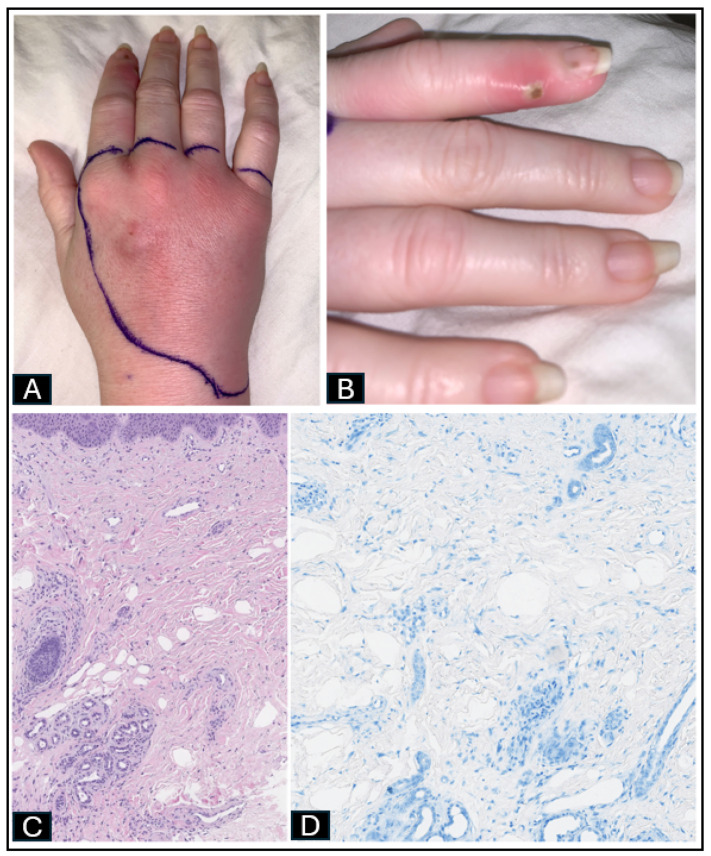
(**A**) Dorsum of the right hand with a 2 × 2 cm non-fluctuating nodule between the second and third metacarpals. (**B**) Pinpoint fluctuant lesion in distal phalanx of the right index finger. (**C**) H&E stain ×10. Sparse interstitial lymphohistiocytic infiltrate with neutrophils and dilated papillary dermal lymphatics. (**D**) AFB stain ×40. No acid-fast bacilli seen.

Her vital signs were blood pressure of 141/67 mmHg, heart rate of 94 bpm, respiratory rate of 18 rpm, and temperature of 37.1 °C. Her initial laboratory workup is summarized in Table 1. Her physical exam revealed an erythematous rash on the dorsum of the right hand with a 2 × 2 cm non-fluctuating nodule between the second and third metacarpals (Figure 1A) and erythema extending to the right index finger with a vesiculopustular lesion in the distal phalanx (Figure 1B). MRI of her right hand showed dorsal right-hand cellulitis with extensor digitorum tenosynovitis and no abscess or osteomyelitis. She was initially treated with intravenous (IV) vancomycin and piperacillin-tazobactam. A skin punch biopsy of the dorsum of the right hand showed mostly dermal fibrosis (Figure 1C) and no microorganisms (Figure 1D). A second biopsy of her right second finger was completed and sent for culture, which grew acid-fast organisms at day 12 of culture. Her antibiotic regimen was changed to clarithromycin 500 mg PO twice a day and ethambutol 800 mg PO daily to empirically cover for possible *Mycobacterium marinum.* Further culture data by MALDI-TOF MS revealed *Mycobacterium chelonae*. Her antibiotic regimen was switched to moxifloxacin 400 mg PO daily and clarithromycin 500 mg PO daily for 6–12 months, along with IV imipenem 500 mg every 12 h (renally dosed) for the first 8 weeks. She was counseled to keep her hands out of the aquarium tank. She agreed to use arm-length gloves and avoid skin abrasions. Her susceptibility culture results showed *M. chelonae* sensitive to clarithromycin (2 mcg/mL), tobramycin (2 mcg/mL), and amikacin (16 mcg/mL); resistant to cefoxitin (>128 mcg/mL), ciprofloxacin (4 mcg/mL), doxycycline (>8 mcg/mL), and trimethoprim-sulfamethoxazole (4/76 mcg/mL); and intermediate to imipenem (8 mcg/mL), linezolid (16 mcg/mL), and moxifloxacin (2 mcg/mL). She was followed at the outpatient infectious disease clinic. She completed 8 weeks of imipenem and 12 months of moxifloxacin and clarithromycin with complete resolution of skin lesions.

Approximately 2 years later, the patient returned to the outpatient clinic for evaluation of exquisitely tender left-hand index finger and distal phalanges necrotic ulcerations over 2 months. Some of the lesions appeared after minor trauma (hang nail, splinter from plants), and others developed spontaneously. She was prescribed doxycycline for possible cellulitis.

**Figure 2:**
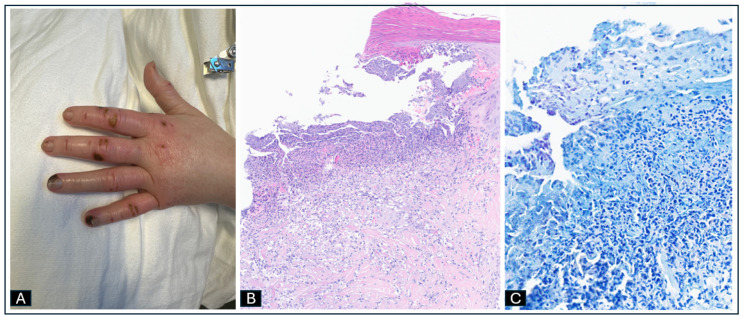
(**A**) Dorsum of the left hand with surrounding erythema and swelling predominant in the left index finger. (**B**) H&E stain ×16. Superficial dermal ulcer with necrosis and brisk superficial neutrophilic inflammation without vasculitis. (**C**) AFB stain ×25. No acid-fast bacilli seen.

Her hand pain worsened, and she was admitted 1 week later to the hospital for further evaluation. Her admission vital signs were blood pressure of 152/78 mmHg, heart rate of 96 bpm, respiratory rate of 19 rpm, and temperature of 36.7 °C. Her laboratory workup is summarized in Table 1. Her physical exam was notable for multiple eschars in the left-hand fingers and dorsum of the left hand, with surrounding erythema and swelling predominant in the left index finger (Figure 2A). MRI of her left hand showed scattered subcutaneous edema of the hand and fingers and early osteomyelitis of the index finger proximal phalanx base. She was initiated on IV vancomycin (renally dosed) and IV ertapenem 1 g daily with a plan to treat for 6 weeks. A skin biopsy of one of the necrotic lesions showed a crusted superficial ulcer with focal occlusive vasculopathy, negative for leukocytoclastic vasculitis (Figure 2B), and negative AFB stain (Figure 2C), gram stain, bacterial and mycobacterial cultures.

**Figure 3:**
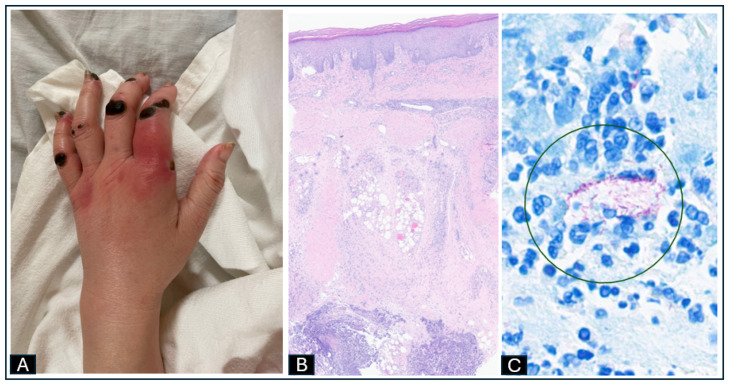
(**A**) Area of swelling and redness in the left index finger with an area of fluctuance at its base. (**B**) H&E stain ×4. Surgical pathology showing subcutaneous abscess with basophilic fat necrosis and dermal fibrosis. (**C**) AFB stain ×80 with clusters of rod-shaped organisms (circle) within the neutrophilic infiltrate.

Approximately 2 weeks later, she developed increased pain, swelling, and redness in the left index finger with an area of fluctuance at the base of the left index finger suspicious for abscess (Figure 3A). She underwent operative debridement and irrigation. Surgical tissue pathology revealed subcutaneous abscess with basophilic fat necrosis and dermal fibrosis (Figure 3B). AFB stain showed clusters of rod-shaped organisms within the neutrophilic infiltrate (Figure 3C). Further cultures were positive for *Candida parapsilosis*. IV caspofungin 70 mg followed by 50 mg daily was added with a plan to complete 3 months and deescalate based on fungal susceptibility results. *Mycobacterium chelonae* also grew at 11 days by MALDI-TOF MS culture. Her antibiotic regimen was switched to clarithromycin 500 mg PO twice a day, doxycycline 100 mg PO twice a day with a plan to treat at least for 4 months, along with IV imipenem 500 mg every 12 h (renally dosed), and to continue caspofungin. Further susceptibility data showed pan-sensitive *C. parapsilosis* and *M. chelonae* sensitive to amikacin (16 mcg/mL), clarithromycin (0.5 mcg/mL), and linezolid (8 mcg/mL); intermediate to imipenem (16 mcg/mL); and resistant to cefoxitin (>128 mcg/mL), ciprofloxacin (>4 mcg/mL), doxycycline (>8 mcg/mL), moxifloxacin (>4 mcg/mL), and trimethoprim-sulfamethoxazole (>4/76 mcg/mL). Her regimen was changed to IV amikacin 25 mg/kg three times a week (the patient had developed hallucinations while on clarithromycin with a similar response to azithromycin, and resolution of hallucinations after macrolide was stopped), imipenem and oral fluconazole 200 mg PO daily for at least 3 months with a plan to continue serial follow up at the infectious disease clinic. Unfortunately, the patient was lost to follow-up.

### 2.2. Case 2: Disseminated M. chelonae

An 81-year-old female patient with a history of myelodysplastic syndrome (on treatment with decitabine), hypogammaglobulinemia (requiring IV immunoglobulin infusions every 4 months), polymyositis on chronic methylprednisolone 4 mg twice a day, chronic non-healing left shin skin ulcer, and two recent hospital admissions for neutropenic fever and left leg cellulitis in the last 2 months presented to the ER for evaluation of fevers, and skin rash in the left lower extremity and right upper extremity or the last 10 days. She had no history of skin trauma.

**Figure 4:**
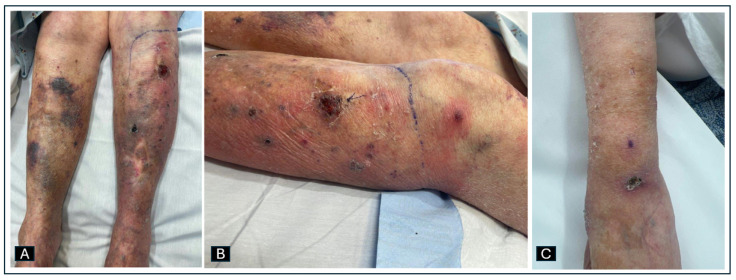
(**A**) Front view of left leg with scattered violaceous-dusky, maculopapular rash. (**B**) Lateral view of left leg with same described rash and large chronic ulcerated necrotic plaque. (**C**) Right upper extremity with scattered plaques with a violaceous center.

Her vital signs were a temperature of 38.1 C, blood pressure of 132/82 mmHg, pulse of 95 bpm, and a respiratory rate of 22 rpm. Her initial laboratory workup is summarized in Table 1. Her physical exam revealed a scattered violaceous-dusky, non-blanchable, maculopapular rash, slightly indurated on her left shin and calf (Figure 4A). On her left shin, there was a large chronic ulcerated necrotic plaque (Figure 4B). On her left thigh and right upper extremity (Figure 4C), she had scattered plaques with a violaceous center.

**Figure 5:**
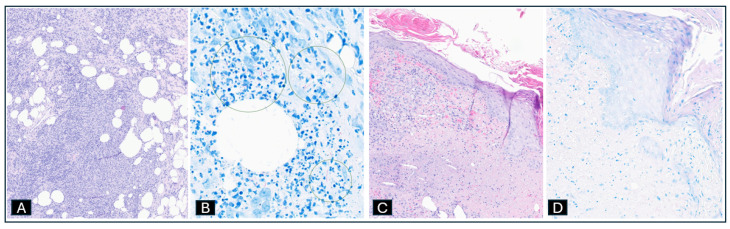
(**A**) H&E stain ×10. Florid neutrophilic lobular panniculitis with granulomatous inflammation. (**B**) AFB stain ×80 numerous rod-shaped organisms (circled) in areas of suppuration within the subcutis. (**C**) H&E stain ×13. Basophilic dermal necrosis with numerous neutrophils with extensive leukocytoclasia and early epidermal necrosis. (**D**) AFB stain ×25. No acid-fast bacilli seen.

She was empirically treated with IV vancomycin 1 g every 12 h, IV cefepime 2 g every 8 h, and posaconazole PO 300 mg twice a day. A skin biopsy taken from one of the lesions in her right upper arm showed neutrophilic lobular panniculitis with granulomatous inflammation (Figure 5A) and numerous rod-shaped organisms in areas of suppuration within the subcutis on AFB stain (Figure 5B). A skin biopsy from the left shin showed basophilic dermal necrosis with numerous neutrophils with extensive leukocytoclasia and early epidermal necrosis (Figure 5C). AFB stain (Figure 5D), gram stain, and humane herpes virus 8 (HHV8) stain were negative.

Despite a negative AFB stain, mycobacterial culture by MALDI-TOF MS from the left leg biopsy grew *M. chelonae*, which indicated a disseminated infection. Empiric treatment was initiated with trimethoprim-sulfamethoxazole PO 800–160 mg every 12 h, azithromycin PO 500 mg daily, and IV eravacycline 50 mg every 12 h. Culture results susceptibilities showed *M. chelonae* susceptible to clarithromycin (0.5 mcg/mL), azithromycin, clofazimine (0.25 mcg/mL), and tigecycline (0.5 mcg/mL); intermediate to amikacin (32 mcg/mL) and tobramycin (4 mcg/mL); and resistant to imipenem (32 mcg/mL), cefoxitin (>128 mcg/mL), ciprofloxacin (>4 mcg/mL), doxycycline (>8 mcg/mL), linezolid (32 mcg/mL), moxifloxacin (>4 mcg/mL), and trimethoprim-sulfamethoxazole (4/76 mcg/mL). Trimethoprim-sulfamethoxazole was stopped. The patient refused to take clofazimine due to concerns of skin discoloration and refused to continue eravacycline or to consider tigecycline due to a history of minor allergic reactions to tetracyclines. She was transitioned to azithromycin PO 500 mg daily, IV imipenem 1 g every 12 h, and IV tobramycin 280 mg 3 times per week. Tobramycin was discontinued due to possible ototoxicity. She continued to be treated with azithromycin and imipenem for the next 7 months, which resulted in complete clinical resolution of skin rash. The patient was followed 2 months after complete recovery at the infectious diseases clinic and regularly over the next 2 years at the oncology clinic without signs of *M. chelonae* infection recurrence.

## 3. Discussion

*M. chelonae* infection is most commonly present as a skin and soft tissue infection (SSTI) but can also present as pulmonary disease, disseminated disease, lymphadenitis, and catheter-associated infections. *M. chelonae* SSTI commonly affects immunosuppressed patients but can also be seen in immunocompetent patients as a refractory to standard antibiotic treatment, chronic skin and/or soft tissue infection following an environmental inoculation after skin trauma, or spontaneously without a clear port of entry [4]. Other risk factors for infection are exposure from cosmetic or plastic surgery procedures, unsterilized acupuncture needles, contaminated tattoo ink, and activities such as pedicure (more commonly seen with *M. fortuitum*), footbaths, and gardening [3,5,6,7,8]. Fish-related activities have been more commonly associated with *Mycobacterium marinum* infections in immunocompetent hosts [3]. However, other NTM such as *M. chelonae* can also cause infections in patients exposed to these activities, as seen in *Case 1*. It is important to recognize significant exposures and have a high index of clinical suspicion to avoid a delayed diagnosis, as the median time between the onset of symptoms and diagnosis is between 2 and 7.9 months [3,9]. Osteomyelitis (as seen in *Case 1*), corneal ulcers, pneumonitis, endocarditis, and lymphadenitis are less common presentations. Tenosynovitis, as seen in *Case 1*, is a rare presentation of *M. chelonae* with few cases reported in the literature [1]. Patients on low-dose systemic corticosteroids and renal transplant patients are at a higher risk of disseminated disease [1,9]. The severity of the infection varies depending on the immune status of the patient. Patients who are immunocompromised and dialysis patients, both seen in *Case 1*, are at a particularly higher risk. NTM and *Candida* sp. coinfections, as the one seen in *Case 1*, are well known in the literature [10]. *Candida* sp. is a common colonizer of the gastrointestinal tract and skin flora and a common opportunistic infection, particularly in immunocompromised hosts.

Constitutional symptoms are rare [2]. Skin lesions do not have a pathognomonic sign but a common sporothrichoid pattern with a variety of lesions, including erythematous or violaceous, tender, papules or plaques, pustules, nodules that slowly spread and later can become ulcers, or abscesses [3,6]. The limbs are more commonly compromised than the head or neck in disseminated disease [1]. However, *M. chelonae* infection in the lower extremities is not common. An extensive review [3] identified 22 cases in the literature since 1948. The most common NTM species identified in renal transplant recipients has been *M. chelonae* (19.1%) [9]. In this population, the risk of losing kidney graft function is 30.2%, and the chance of dying is up to 20.9% from the infection itself or other complications such as sepsis, cardiac arrest, and respiratory failure [9].

Dermatopathology findings are non-specific and dependent on the infection stage and immune status of the host, which is a weak-to-absent granulomatous pattern that is more common in immunocompromised patients. The most common pathology finding is a mixed diffuse or granulomatous inflammatory infiltrate neutrophil predominant with or without dermal or subcutaneous abscesses and acute or chronic panniculitis. Acid-fast bacilli (AFB) stains, gram stains, and routine cultures are usually negative and most likely to be positive in immunocompromised patients [1]. Routine bacterial cultures do not identify mycobacteria; therefore, mycobacterial culture with prolonged culture is key for diagnosis [2].

Differential diagnosis includes infectious and non-infectious etiologies. Fungal infections such as blastomycosis, coccidiomycosis, paracoccidiomycosis, cryptococcosis, other granulomatous infections such as cat-scratch disease, nocardiosis, leishmaniasis, and other atypical mycobacterial infections (tuberculosis, leprosy) may be considered. Non-infectious etiologies include sarcoidosis, superficial granulomatous pyoderma, ruptured cysts, and follicles.

*M. chelonae* treatment is usually prolonged, which increases the chance of toxicity and antimicrobial resistance. Antibiotic monotherapy is not recommended, given the risk of antibiotic resistance. The American Thoracic Society/Infectious Diseases Society of America (ATS/IDSA) recommends an oral macrolide combined with cefoxitin, amikacin, or imipenem for the initial treatment of NTM [11]. Susceptibility testing may take 4–6 weeks and should include amikacin, doxycycline, imipenem, fluoroquinolones, cefoxitin, clarithromycin, and a sulfonamide [12]. Imipenem is a good alternative to species resistant to cefoxitin. Oral options include clarithromycin, omadacycline, clofazimine, ciprofloxacin, and doxycycline [12]. Clarithromycin is usually used in initial regimens due to high oral bioavailability, gastrointestinal tolerance, and good tissue penetration [3]. Omadacycline, a new semisynthetic third-generation tetracycline, available in oral and IV presentation, has fewer side effects and higher activity against *M. chelonae* than other standard tetracyclines [13]. Clofazimine has shown excellent activity against rapid growth mycobacteria (susceptibility rate 100% for *M. chelonae* in a Japanese study), synergy with amikacin, and it is considered an interesting option given long half-life (65–70 days), and high concentration in macrophages and phagocytes [14]. Bedaquiline, a diarylquinoline, is an anti-tuberculous medication used in severe infections, which seems to be a promising medication to treat extrapulmonary NTM infections [15]. Tigecycline is only available intravenously and has a high rate of nausea and vomiting [14]. At least 4 months of treatment is needed for limited skin and soft tissue infection, and 6–12 months with 2–3 agents (IV therapy initially) if severe or disseminated disease is present [3,14]. There may be different susceptibility patterns even within the same *M. chelonae* isolated species along the course of treatment or even after recovery, so it is particularly important to complete susceptibility testing in all clinical isolates. A combination of medical and surgical therapy is usually required, especially in immunocompromised patients. However, antibiotics alone may be preferred in selected cases. A systematic review of NTM hand infection showed that 86.5% of patients required at least one surgical debridement, and approximately 32.4% required multiple surgeries [16]. Continuous follow-up is important to evaluate adverse drug reactions and clinical responses until there is a complete resolution. In transplant patients, it is important to consider reducing or withdrawing immunosuppressive agents during treatment.

New therapies for *M. chelonae* include bacteriophage therapy [6], which consists of using organisms that are ubiquitous in the environment to infect and kill bacteria. This type of novel treatment has been useful for the treatment of severe *M. chelonae* infections in conjunction with traditional antibiotic therapy and surgery. Limitations of bacteriophage therapy are availability, regulatory challenges, development of phage resistance, complex interaction with the immune system of the host, and development of neutralizing antibodies, which have unclear clinical consequences.

## 4. Conclusions

It is important to increase awareness among clinicians about *M. chelonae* as an uncommon etiology of skin and soft tissue infections. Having a high clinical index of suspicion for *M chelonae*, especially in non-healing skin lesions and/or infections resistant to routine antibiotics and including mycobacterial cultures and AFB stain, are crucial to have an early diagnosis and prevent severe disease with high mortality, especially in renal transplant patients.

## Figures and Tables

**Table 1:** Summary of laboratory workup *Case 1* (first admission), *Case 1* (second admission), and *Case 2*. CRP: C-reactive protein. ANC: Absolute Neutrophil Count. Normal values: white blood cell count 3.4–9.6 × 10^9^/L, ANC 1.56–6.45 × 10^9^/L, hemoglobin 11.6–15 g/dL, platelet count 157–371 × 10^9^/L, creatinine 0.59–1.04 mg/dL, CRP ≤ 8.0 mg/L.

Test/Case	Case 1 (First Admission)	Case 1 (Second Admission)	Case 2
White blood cell count (×10^9^/L)	10	8.4	0.9 (ANC: 130)
Hemoglobin (g/dL)	10.1	8.5	7.6
Platelet count (×10^9^/L)	323	243	21
Creatinine (mg/dL)	2.04	5.74	
Other blood workup	CRP 10.5 mg/L. Blood cultures no growth	CRP 34 mg/L	Blood and fungal cultures no growth. CRP 91.9 mg/L

## Data Availability

The data presented in this study are available on request from the corresponding author.
